# Detection, Analysis and Clinical Validation of Chromosomal Aberrations by Multiplex Ligation-Dependent Probe Amplification in Chronic Leukemia

**DOI:** 10.1371/journal.pone.0015407

**Published:** 2010-10-25

**Authors:** Adam Abdool, Amber C. Donahue, Jay G. Wohlgemuth, Chen-Hsiung Yeh

**Affiliations:** Department of Hematology and Oncology, Quest Diagnostics Nichols Institute, San Juan Capistrano, California, United States of America; Health Canada, Canada

## Abstract

Current diagnostic screening strategies based on karyotyping or fluorescent in situ hybridization (FISH) for detection of chromosomal abnormalities in chronic lymphocytic leukemia (CLL) are laborious, time-consuming, costly, and have limitations in resolution. Multiplex ligation-dependent probe amplification (MLPA) can simultaneously detect copy number changes of multiple loci in one simple PCR reaction, making it an attractive alternative to FISH. To enhance the clinical robustness and further harness MLPA technology for routine laboratory operations, we have developed and validated a protocol for comprehensive, automatic data analysis and interpretation. A training set of 50 normal samples was used to establish reference ranges for each individual probe, for the calling of statistically significant copy number changes. The maximum normal ranges of 2 and 3 standard deviations (SD) are distributed between 0.82 and 1.18 (Mean ± 2SD, 95% CI, *P* = 0.05), and between 0.73 and 1.27 (Mean ± 3SD, 99% CI, *P* = 0.01), respectively. We found an excellent correlation between MLPA and FISH with 93.6% concordance (*P*<0.0001) from a testing cohort of 100 clinically suspected CLL cases. MLPA analyses done on 94/100 patients showed sensitivity and specificity of 94.2% and 92.9%, respectively. MLPA detected additional copy number gains on 18q21.1 and chromosome 19, and novel micro-deletions at 19q13.43 and 19p13.2 loci in six samples. Three FISH-failed samples were tested positive by MLPA, while three 13q- cases with a low percentage of leukemia cells (7%, 12% and 19%) were not detected by MLPA. The improved CLL MLPA represents a high-throughput, accurate, cost-effective and user-friendly platform that can be used as a first-line screening test in a clinical laboratory.

## Introduction

Advances in cancer genomics allow us to determine and quantify disease-associated genetic profiles, and to improve clinical diagnosis/prognosis, tumor classification and ultimately, cancer therapy [1]. Chromosomal alterations in leukemia have been shown to have prognostic and predictive value, and are also important markers of minimal residual disease in the follow-up of leukemia patients [2]. The complex process that drives the development of leukemia could rise from several clonal molecular abnormalities, including copy number gains and losses in the genome leading to activation of proto-oncogenes and silencing (or deletion) of tumor suppressor genes, respectively [3], [4]. Chronic lymphocytic leukemia (CLL) is the most common adult leukemia in developed countries. Specific chromosome copy number alterations characteristic of CLL, such as loss of the 13q14 region (with a frequency of 50–60%), trisomy of chromosome 12 (15–25%), and deletions of 11q22 (10–20%) and 17p13 (5–10%), have been shown to provide clinically relevant prognostic information and help identify more aggressive disease [5], [6]. Patients with leukemia cells positive for deletion of 17p13 or 11q22 have an inferior prognosis compared with normal karyotype or del(13q14), and appear to be resistant to standard chemotherapy regimens [5]–[7]. Trisomy 12 has been associated with an intermediate-to-unfavorable prognosis [6], [7]. Unlike other hematological malignancies, chromosome translocations are relatively rare in CLL [8].

Conventional metaphase karyotyping detects chromosomal abnormalities in only 40–50% of CLL cases, because obtaining mitoses representing malignant cells is problematic due to the low mitotic activity of CLL cells in vitro, even with mitogen stimulation [9]. Fluorescent in situ hybridization (FISH), which uses labeled probes targeted to the most commonly altered genomic regions, has proven to have higher resolution than traditional cytogenetics. FISH enables detection of alterations in interphase nuclei and metaphase chromosomes and can reveal abnormalities in 80% of cases [5]–[7]. In clinical practice, however, current FISH analysis is only capable of detecting deletions or amplifications of sequences larger than 20–50 kb [10], and due to the high cost of these labeled probes, FISH testing is generally restricted to 13q14, TP53, ATM and chromosome 12 for CLL cases. More recently, microarray-based comparative genome hybridization (array-CGH) and high-density SNP arrays allow high resolution genome-wide scans for detection of copy-number variations (CNVs) in a single hybridization [11], [12]. Although global array-based approaches can provide high resolution data on CNVs in individuals, these methods are limited by low throughput, high cost, and a long turnaround time, and there remains a need for simple, cost-efficient methods to screen chromosomal alterations across larger populations.

The PCR-based multiplex ligation-dependent probe amplification (MLPA) technique for gene dosage determination offers a higher throughput, less labor intensive alternative. By comparison of the abundance of a region of interest in a CLL patient's cells to that of a cohort of normal individuals, abnormalities in the number of copies of each DNA sequence can be identified and quantified. The introduction of universal primers in MLPA not only makes multiplex target detection much easier and consistent, but also significantly cuts down the cost [13], [14]. Nevertheless, detection accuracy is of particular importance if diagnostic laboratories wish to augment or replace multiprobe FISH with MLPA. The adoption of this technique will certainly depend on the robustness of the MLPA technique and the analysis algorithm used, in order to avoid missed or miscalled results. Here we report the clinical application of MLPA for the detection of common genomic deletions and trisomies associated with CLL prognosis, in direct comparison to FISH. The new strategy we developed for MLPA data analysis is robust, automated, consistent and cost-effective. It is our recommendation to establish just such an analysis protocol to incorporate quality checks, include a reference cutoff range for each probe, and provide unequivocal scoring criteria for accurate interpretation of MLPA results. This is the first report to define these analytical and interpretative parameters for MLPA application in CLL prognostics.

## Materials and Methods

### Patient Samples

All patient samples were collected with a signed informed consent in accordance with Quest Diagnostics Nichols Institute's Institutional Review Board (IRB)-approved protocol. Fifty peripheral blood samples from healthy donors were used as the training set for the establishment of MLPA analytical and interpretative parameters. One hundred referrals for CLL panel testing by FISH were re-screened using MLPA. The FISH patient samples were tested as part of the cytogenetics diagnostic service at Quest. Patient identifiers were removed and replaced by a numbering scheme allowing all MLPA analysis performed without prior knowledge to any abnormalities identified by FISH. After MLPA analysis was completed, all data were decoded to determine concordance between FISH and MLPA and frequency of each chromosomal abnormality.

### Multiplex Ligation-Dependent Probe Amplification (MLPA)

#### DNA extraction

DNA was extracted from blood samples received in lavender (purple)-top tubes containing ethylenediaminetetraacetic acid (EDTA) using the BioRobot EZ1 Workstation (Qiagen, Valencia, CA, USA) following the manufacturer's instructions. All isolated DNA was quantified by NanoDrop spectrophotometry (NanoDrop, Wilmington, DE, USA).

#### MLPA assay

Genomic DNA samples (75–200 ng) were subjected to PCR reactions containing MLPA P038-A2 CLL probemix-2, SALSA PCR reagents and polymerase (MRC-Holland, The Netherlands) following hybridization and ligation steps. All tests were performed in duplicate in an ABI 9700 PCR instrument, and amplified PCR products were analyzed by GeneMapper software v4.0 on an ABI3730 capillary sequencer (Applied Biosystems, Foster City, CA, USA). The MLPA assay was performed according to the manufacturer's protocol, with the following exceptions: (i) DNA denaturation was done at 98°C for 15 min to increase efficiency, especially for GC-rich sequences; (ii) 40 PCR cycles were performed; (iii) At least five normal control samples and one negative control were included for each MLPA run.

#### MLPA data analysis

The calculation of probe ratios consisted of a mathematical comparison between relative quantities of target DNA amplified from a test patient sample, to those generated in a normal control sample. Analysis of probe ratios from a pool of 50 normal control samples allowed us to determine statistical variation within the normal range, and to assign confidence limits (or standard deviations, SD) to each diagnostic probe to be called deletions or amplifications. To streamline the generation and manipulation of probe ratio data, unique automated Excel spreadsheet was devised and written for this purpose. The peak area data from GeneMapper software were imported into a spreadsheet-based automated analysis system. The system contained a series of quality checks to ensure that samples failing to pass quality checks will be flagged and not to be reported. These checks ensure that diagnostic peaks for input DNA amount, denaturation, hybridization, ligation were in range, and signal sloping was corrected in both normal and patient samples.

For a test patient sample, a series of ratios was generated for each diagnostic probe against 13 internal reference probes, rather than generating a single ratio for each probe by using an average of reference probes, to minimize differential tail-off effect. Similarly, on each run, probe ratios were also calculated for each of at least five normal controls, and the mean of these ratios formed the denominator in the formula. All probe ratios in normal controls should not exceed ±2 SD of normal range to be considered valid. The “median” value of a total of 13 normalized ratios (Patient sample: Normal control) gave the final result for each individual diagnostic probe:
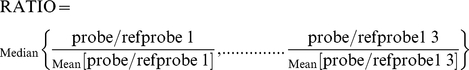



Each probe was called “deleted”, “normal” or “amplified” depending on whether the ratio fell within or outside of the established normal range (mean ± 2SD or mean ± 3SD). If more than 50% of the probe ratios in a particular region indicated a deletion or amplification, the result for that chromosome region was called as abnormal. Finally, our spreadsheet-based analysis system produced a summary table listing each MLPA probe in each test sample, with color highlighting to indicate a deletion (Red) or amplification (Blue).

### Fluorescence in situ hybridization (FISH)

Samples of peripheral blood from suspected CLL patients were analyzed with a FISH panel at Quest Diagnostics, using the following Vysis probes: ATM (11q22.3), p53 (17p13.1), D13S319 (13q14.3)/D13S1020 (13q34), and D12Z3 (CEP 12) (Abbott Molecular, Abbott Park, IL, USA), according to the manufacturer's instructions. Dual-color fluorescent signals were visualized under fluorescence microscopy, and for each sample, at least 300 interphase nuclei were analyzed and scored by two independent investigators. The cutoff values for each individual probe on peripheral blood and bone marrow samples are: 11q- (6%)/(7%), +12 (3%)/(3%), 13q- (6%)/(6%), 17p- (10%)/(8%), respectively.

### Statistical Analysis

The significance of any change in DNA copy number for MLPA, and data from MLPA and FISH, were compared with Fisher's exact test and nonparametric tests as appropriate. All probabilities were 2-tailed, and *P* values <0.05 were considered statistically significant.

## Results

### Establishment of Normal Range for Each Individual MLPA Probe

In typical CLL MLPA reactions, mixtures composed of up to 40 probes (13 reference and 27 diagnostic) can be used, which makes it easy to quantitatively assess the copy number changes of different chromosome regions simultaneously. Dissimilarities in PCR efficiency between different probes, and probe-to-probe and sample-to-sample variations are known factors impacting reference ranges, therefore a common theoretical or arbitrary range for all probes is inappropriate. Instead of using an arbitrary ratio range (e.g., 0.75–1.25, 0.8–1.2 or 0.95–1.05) as a single universal cutoff value for all probes, which is applied in most MLPA studies [13], [14], we set out to establish a normal range for each MLPA probe to provide a more appropriate baseline from which any copy number variation (CNV) will be confidently identified in CLL patients. Our reference ranges were derived from a training pool of 50 healthy subjects. Each reference range of the 27 diagnostic probes from one sample was calculated against the other 49 normals. The reference range data showed a normal distribution in each case, and a narrow variation in the mean and SD between these normals, with the Mean ± 2SD value ranging at maximum from 0.82 to 1.18 (95% CI, *P* = 0.05), and Mean ± 3SD value from 0.73 to 1.27 (99% CI, *P* = 0.01) (Table 1). For optimal identification of CNVs, we recommend the use of at least 5 normal genomic DNA samples as controls in each run, instead of using pooled genomic DNAs because CNVs in each individual of the pool will become averaged. As a result, it will reduce the chance to detect real chromosomal aberrations and therefore a genomic imbalance by MLPA. Further, each MLPA application should establish its own normal reference range for each probe, and a standardized methodology should be used for data computation and analysis. Standardizing MLPA interpretation in this way should improve the consistency and accuracy of CNV detection.

**Table 1:** Normal reference range established for each individual probe for CLL MLPA.

Probe (gene/chromosome)	Normal range (Mean ± 2SD; 95% CI, *P* = 0.05)*	Normal range (Mean ± 3SD; 99% CI, *P* = 0.01)*
**PTEN1 10q23.3**	0.84–1.16	0.76–1.24
**PTEN2 10q23.3**	0.89–1.11	0.84–1.16
**ATM1 11q23**	0.85–1.15	0.78–1.22
**ATM2 11q23**	0.87–1.13	0.80–1.20
**ATM3 11q23**	0.84–1.16	0.76–1.24
**ATM4 11q23**	0.88–1.12	0.82–1.18
**RDX 11q23**	0.91–1.09	0.86–1.15
**CD27 12p13.31**	0.82–1.18	0.73–1.27
**APAF 12q23.1**	0.86–1.14	0.83–1.17
**IGF1 12q23**	0.87–1.13	0.85–1.15
**PAH1 12q23**	0.89–1.11	0.83–1.17
**PAH2 12q23**	0.88–1.12	0.82–1.18
**RB1 13q14.2**	0.87–1.13	0.81–1.19
**KCNRG 13q14.3**	0.89–1.11	0.84–1.16
**DLEU1 1 13q14.3**	0.87–1.13	0.80–1.20
**DLEU1 2 13q14.3**	0.87–1.13	0.80–1.20
**DLEU1 3 13q14.3**	0.85–1.15	0.78–1.22
**ATP7B 13q14.3**	0.87–1.13	0.80–1.20
**TP53 1 17p13.1**	0.83–1.17	0.74–1.26
**TP53 2 17p13.1**	0.83–1.17	0.81–1.19
**TP53 3 17p13.1**	0.83–1.17	0.81–1.19
**TP53 4 17p13.1**	0.84–1.16	0.76–1.24
**SMAD4 18q21.1**	0.86–1.14	0.79–1.21
**CDKN2D 19p13.2**	0.86–1.14	0.80–1.20
**LDLR 19p13.2**	0.89–1.11	0.83–1.16
**CCNE1 19q12**	0.89–1.11	0.83–1.17
**CHMP2 19q13.43**	0.89–1.11	0.83–1.17

*Both 95% and 99% confidence interval corresponding to normal DNA content of all probes are presented. These data were based on 50 DNA samples of blood from healthy controls.

### Chromosomal Aberrations Detected by MLPA

**Figure 1:**
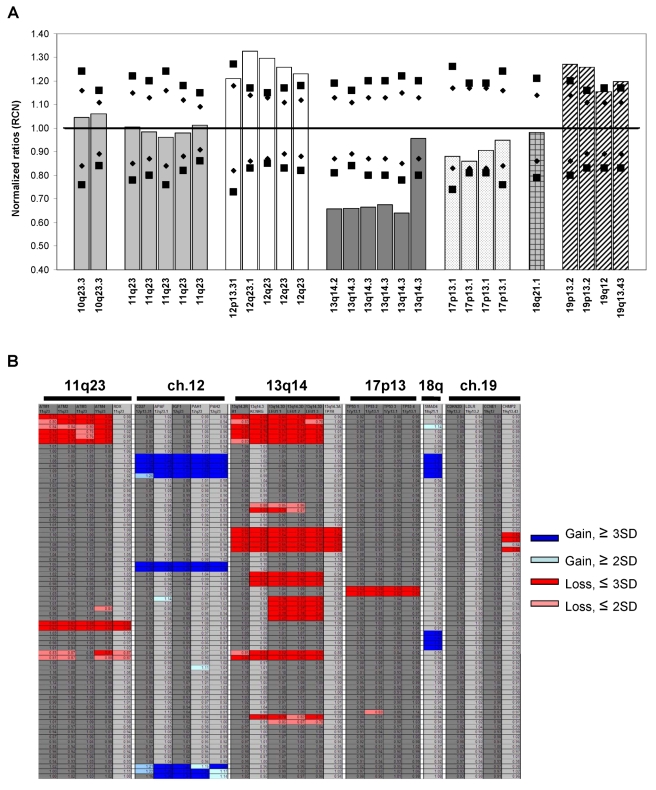
Representative MLPA analysis on CLL patients. (**a**) Normalized ratio plot (relative copy number, RCN) from a CLL patient with trisomy 12 and 19, and 13q14 deletions. The data were normalized to those of normal controls. The reference ranges of 2 SD (Mean ± 2SD, 95% CI, *P* = 0.05) and 3 SD (Mean ± 3SD, 99% CI, *P* = 0.01) for each probe are shown by diamonds and squares, respectively. (**b**) MLPA analysis of copy number changes on multiple CLL samples are called and highlighted on a heatmap as blue (gain, ≥3SD), light blue (gain, ≥2SD), red (loss, ≤3SD) and pink (loss, ≤2SD) blocks that lie outside of reference ranges of each probe. Note that the high resolution of MLPA in several 13q- and 11q- cases can pinpoint deletion region down to a single gene level.

A testing set of 100 samples from suspected CLL patients was analyzed for chromosomal abnormalities by MLPA. MLPA probes target regions commonly associated with CLL prognosis, such as 13q14, ATM, and TP53, and trisomy 12. Additionally, the probe mix also targets other genomic loci with imbalances in CLL, e.g., 10q23, 18q21 and chromosome 19 [15]. Figure 1 shows a representative bar graph (A) and a heatmap (B) of relative copy number changes in CLL patients by MLPA. Although peripheral blood samples were not subjected to B-cell purification before the MLPA assay, cases with low percentages of cells carrying genomic alteration can be reliably detected and scored by our analysis. The key criteria and guidelines for final call on chromosome copy number loss or gain are detailed in Table 2. In the validation samples, deletions in 13q14 with >20% of cells carrying this aberration, deletions in 11q23 with >12% abnormal cells, or trisomy 12 with at least 25% leukemia cells were detected and called by our system. In addition, multiallelic abnormalities from the same patient, e.g., 13q-/11q-, 13q-/17q-, 13q-/19q13.43-, 12tri/17p- or 12tri/13q-/19+ were identified. In 52 of the 100 testing samples (52%), chromosomal abnormalities were detected by MLPA: 25 cases (25%) showed loss of the 13q14 region; 8 cases (8%) showed trisomy of chromosome 12; 3 cases (3%) showed loss of the ATM gene; 3 cases (3%) showed loss of the TP53 gene; and 13 cases (13%) showed multiallelic imbalance (Table 3). Overall, a good correlation was found between MLPA and FISH results, and all abnormalities observed by FISH were also identified by MLPA, with the exception of three 13q- cases with a prohibitively low percentage of leukemia cells (7%, 12% and 19% by FISH).

**Table 2:** MLPA test for CLL: interpretative criteria and calling guidelines.

Normalized probe ratio	Calling guidelines	Interpretation, *p*-value
Within Mean ± 2SD	Duplicates show > = 50% probes in the cluster are within this range	Normal
Out of Mean ± 3SD	Duplicates show > = 1 probe in the cluster are out of this range	Deletion or Amplification, *P*<0.01
Between Mean ± 2SD and Mean ± 3SD	Duplicates show > = 50% probes in the cluster are within this range	Loss or Gain, *P*<0.05

**Table 3:** Frequency of genomic alterations detected for each chromosome in 100 suspected CLL cases by MLPA and FISH.

Genomic alterations	MLPA cases (%)	FISH cases (%)
13q-	25 (25)	26 (26)
12 Trisomy	8 (8)	8 (8)
11q-	3 (3)	3 (3)
17p-	3 (3)	3 (3)
18q+	1 (1)	NA
19q13.43−	1 (1)	NA
19p13.2−	1 (1)	NA

Multiallelic alterations		
13q-/11q-	6 (6)	6 (6)
13q-/17p-	3 (3)	3 (3)
13q-/19q13.43−	2 (2)	NA
13q-/12tri/19+	1 (1)	NA
12tri/17p-	1 (1)	1 (1)

Total cases include cases detected by FISH or by MLPA.

NA, not applicable.

### MLPA Detects Genomic Abnormalities Not Identified by FISH

It is noteworthy to mention that in six of the CLL samples tested, MLPA detected additional copy number gains on 18q21.1 and chromosome 19, and novel micro-deletions at 19q13.43 and 19p13.2 loci, which FISH probes did not cover (Table 3). Most importantly, the higher resolution of MLPA was demonstrated in several 13q- and 11q- cases, in which MLPA was able to pinpoint deletion of a small region down to a single gene level, for example, a small deletion that was only detected in the DLEU gene on 13q14.3, but not the adjacent RB1, KCNRG or ATP7B genes. Another example was a deletion only detected in the ATM gene of 11q23, but not the adjacent RDX genes (Fig. 1B). Our improved technique should allow the detection of small submicroscopic losses or gains that FISH will miss.

### Concordance between MLPA and FISH

A total of 52 chromosomal alterations for CLL were detected using both techniques. MLPA and FISH data were in agreement in 93.6% of cases (88/94), and the failure rate was 5–6% (3/52) for both methods (Table 4). Detection of alterations was similar using either MLPA or FISH (with 93.6% concordance, P<0.0001). MLPA analyses done on these patients showed sensitivity and specificity of 94.2% and 92.9%, respectively. Discordant results were found in six samples: three patients with a low percentage of cells (<20%) carrying 13q14 deletion was detected by FISH, but not by MLPA; three FISH-failed samples were detected positive by MLPA.

**Table 4:** Concordance of MLPA with FISH on 94 suspected CLL specimens.

	MLPA, n (%)				
FISH	Positive	Negative	Total	Concordance	*P* *
Positive	49 (94.2)	3 (5.8)	52	93.6%	<0.0001
Negative	3 (7.1)	39 (92.9)	42	93.6%	<0.0001

*All comparisons used two-tailed Fisher's exact test.

### Sensitivity of CLL MLPA

**Figure 2:**
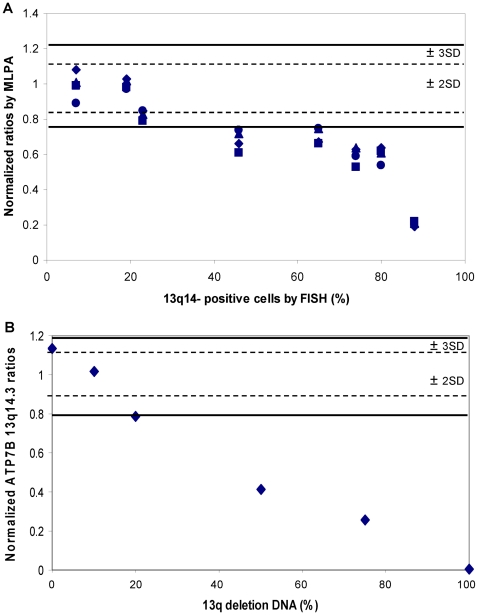
Correlation between MLPA and FISH analyses on 13q14- samples. (**a**) Normalized ratios by MLPA were grouped by the percentage of FISH-identified leukemia clones carrying 13q14 deletion. The distribution of MLPA ratios showed that 13q14- cells must represent at least 20% (if using 2SD as cutoff) or 40% (if using 3SD as cutoff) of the total population in a sample to be called a statistically significant copy number change. (**b**) Mixing study using a homozygous 13q- DNA spiked into normal control DNA to create samples containing 0, 10%, 20%, 50%, 75% or 100% alterations. This study also showed a 20% detection limit on 13q deletion by MLPA.

Because different CLL blood and bone marrow samples, depending on the disease state, contain different percentage of leukemia cells, the results of normalized ratios from MLPA were segregated by percentage of FISH-positive leukemia cells. In the 13q14 deletion cases, as the portion of tumor population increased, the MLPA ratios gradually moved out of the normal ranges of ±2 or 3 SD. Leukemia clones with 13q14 deletion have to make up at least 20% (2SD) or 40% (3SD) of the total population in a sample to be called statistically significant copy number change (Fig. 2A). A mixing study to determine MLPA sensitivity (or limit of detection) was also performed. In these experiments, DNA samples with a homozygous genetic abnormality (e.g., 13q-) were spiked into normal control DNA to generate testing materials with 0, 10%, 20%, 50%, 75% or 100% alterations. Figure 2B shows that 13q deletion can be reliably detected and called by our MLPA protocol if they are present in at least 20% of the total DNA consistent with our previous findings. Moreover, it demonstrates that the reference ranges for each MLPA probe to distinguish between normal and abnormal, the interpretative criteria, and the calling guidelines have been set correctly.

## Discussion

The present work details a novel comprehensive MLPA platform for mapping and assessing the significance of chromosomal abnormalities in CLL. MLPA can provide detailed multiplex profiles of chromosomal aberrations in tumor samples in a relatively short period of time. MLPA data analysis and interpretation are critical for calling real amplification or deletion events in each chromosome region. In contrast to most MLPA studies that applied only a handful normal samples in each experiment or used arbitrary cutoff ratios (subjective values), our report represents the first study to (i) develop and validate a vigorous analytical and scoring criteria for MLPA to robustly map chromosomal aberrations in CLL, with parameters adapted to the characteristics of individual probes, (ii) use reference range of each probe, Mean ± 2SD and ± 3SD (95% CI, *P* = 0.05 and 99% CI, *P* = 0.01, respectively), to determine statistically significant copy number variation (CNV), (iii) apply a stringent calling guideline to cover equivocal cases in which the normalized probe ratios fell between 2SD and 3SD borderlines.

The reference range is generated by randomly selecting a training subset of 50 healthy individuals, and produces consistent normal ratios for the determination of genomic imbalance. We further reduced probe-to-probe and sample-to-sample variations by segmenting the 13 internal reference probes. This process correlates 27 diagnostic probes across all 50 normals, assigning the arithmetic mean and SD of the normalized ratios for each individual probe to produce highest accuracy in individual event calls. A large amount of information is encoded by original probe ratio data, and the reference range is thus established to reduce that information content to a minimal set of discrete gains, losses, or neutral copy numbers. Observation of variation within the control sample pool has allowed us to evaluate performance of the MLPA method, and optimize application of the technique in patients with CLL.

Each chromosomal alteration presents different analytical challenges, not only in dynamic range, but also in their noise characteristics, which is often overlooked. For example, there are challenges unique to allelic loss in CLL. First, deletion is restricted in its size, and second, only two copies of a locus can be lost. This is different from amplification. The lacking of real magnitude and interference from normal DNA in the sample, making it difficult to make a deletion call, and this is further exacerbated for single-copy events at the margins of signal and noise. The limit of detection (LOD) of our improved CLL MLPA assay for calling an allelic loss is approximately 20% of that leukemia clone circulating in the bloodstream. Although the sensitivity is somewhat lower than the sensitivity obtained with interphase FISH (5–10%), such detection level is sufficient for most untreated CLL patients at diagnosis (2, 6). On the other hand, absence of an allelic copy is readily detected while gains in copy number are more problematic to confirm by FISH, especially if the distance between the probes is small [16]. This is a key difference between the methods, with MLPA having the potential to more accurately identify and quantify copy number gains.

To adapt to the diversity of variation among individual probes, samples and alterations, we developed and validated a multi-component scoring scheme for the detection of copy-number changes on a large repository of suspected CLL samples. MLPA produced strong concordance (93.6%) with the gold standard, FISH, without pre-enrichment of malignant B-cells, further enhancing its clinical utility. Fourty-nine abnormalities identified by MLPA were previously reported deletions and trisomy. Six abnormalities were not covered by a standard FISH probe panel. Among these, CLL MLPA analysis identified a complex amplification on 18q21.1 containing the *SMAD4* gene, and a gain (trisomy) of chromosome 19, as well as small intragenic deletions at the 19q13.43 *CHMP2* and 19p13.2 *CDKN2D* loci. Trisomy 19 is a CLL-associated genomic abnormality observed in ∼5% of CLL cases [15], [17]. One of our samples with trisomy 19 also carried a trisomy of chromosome 12 and a loss of the 13q14 region. Co-existence of these three aberrations, as well as trisomy 12/19, have been documented [17], [18]. The *SMAD4* gene, a member of the *MAD* gene family, is involved in TGF-beta signal transduction. Overexpression of *SMAD4* (by 18q21.1 amplification) could thereby play a role during the development of CLL resistance to TGF-beta [19], [20]. *CDKN2D* gene (cyclin-dependent kinase inhibitor 2D) on 19p13.2 belongs to the *INK4* family. Members of the *INK4* family play widespread and independent roles in tumor suppression in a variety of cancers [21]. *CHMP2* gene on 19q13.43 belongs to the chromatin-modifying protein/charged multivesicular body protein family. The gene product is involved in degradation of surface receptor proteins and formation of endocytic multivesicular bodies and is required for regulation of cell cycle progression [22]. Overall, MLPA has the potential to identify more abnormalities from a single sample than FISH.

Our automated CLL MLPA data processing, analysis and interpretation strategy has significant clinical advantages, especially when handling large MLPA data sets, when samples are of different quality, and when interpretation of MLPA electropherograms is too complex. Additionally, for tests that could be applied in the diagnostic setting, turnaround time is a critical factor. With MLPA, the total process-to-report time, including data analysis, is 2–3 days compared to 7–10 day for FISH. MLPA is also cheaper and less labor intensive compared with FISH. In summary, our improved MLPA offers the advantages of multiplexing, high-throughput, high resolution and low-cost for detection of copy number changes over classical karyotyping/FISH in routine CLL diagnostics.
